# Supplementary material for: Impact of solvent forces and broken symmetry on the assembly of designed proteins at a liquid-solid interface
Source: Nat Commun. 2026 Mar 13;17:2446. doi: 10.1038/s41467-026-69170-0 (PMC12987978; doi:10.1038/s41467-026-69170-0)
Supplement: Supplementary file 1 — Supplementary Info [file 41467_2026_69170_MOESM1_ESM.pdf]

Supplementary Information

Impact of solvent forces and broken symmetry on the assembly of designed proteins at a liquid-solid interface

Sakshi Yadav Schmid[1,2†‡§], Benjamin Helfrecht[1†], Amy Stegmann[3†], Benjamin A. Legg[1], Harley Pyles[4,5], Jiajun Chen[2#], John R. Edison[6**], Maxim Ziatdinov[1,7], Zdenek Preisler[6††], Orion Dollar[8‡‡], Stephen Whitelam[6], Sergei Kalinin[7,9], David Baker[4,5,10], Christopher J. Mundy[1,8,*], Shuai Zhang[1,2,*] and James J. De Yoreo[1,2,*]

[1]Physical Sciences Division, Pacific Northwest National Laboratory, Richland, WA 99354, USA. [2]Materials Science and Engineering, University of Washington, Seattle, WA 98105, USA. [3]Molecular Engineering and Science Institute, University of Washington, Seattle, WA 98105, USA. [4]Department of Biochemistry, University of Washington, Seattle, WA 98195, USA. [5]Institute for Protein Design, University of Washington, Seattle WA 98105, USA. [6]Molecular Foundry, Lawrence Berkeley National Laboratory, California 94720, USA. [7]Center for Nanophase Materials Sciences, Oak Ridge National Laboratory, Oak Ridge, Tennessee 37831, USA, [8]Department of Chemical Engineering, University of Washington, Seattle, WA 981915, USA. [9]Department of Materials Science and Engineering, University of Tennessee, Knoxville, TN 37996, USA. [10]Howard Hughes Medical Institute, University of Washington, Seattle, WA 98105, USA.

†These authors contributed equally.

*Corresponding author email: james.deyoreo@pnnl.gov (JJDY); shuai.zhang@pnnl.gov (SZ); chris.mundy@pnnl.gov (CJM)

Current addresses:

‡ Institute of Materials and Interfaculty Bioengineering Institute, Ecole Polytechnique Fédérale de Lausanne (EPFL), 1015 Lausanne, Switzerland.

§National Center of Competence in Research Bio-Inspired Materials, University of Fribourg, Chemin des Verdiers 4, 1700 Fribourg, Switzerland.

Mattson Technology, Fremont, CA 94538, USA

**Department of Chemical and Biomolecular Engineering, Johns Hopkins University, Baltimore, Maryland 21218, USA

††CNR-ISPC, Via Biblioteca 4, 95124 Catania, Italy

‡‡Flagship Pioneering, Cambridge, MA 02142, USA

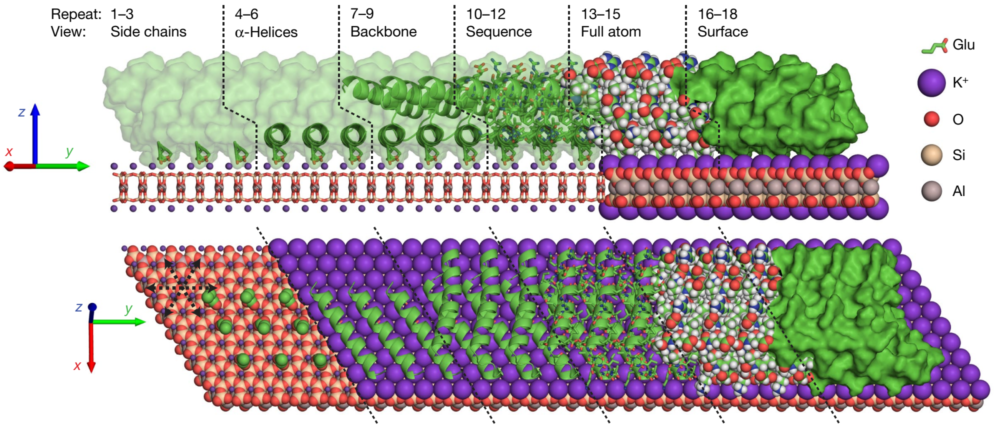

Supplementary Figure 1: Model of DHR10-mica18 protein lattice-matched to mica (001) surface through the K+ sublattice. Repeats 1–3 illustrate the lattice-matched glutamate (Glu) side chains. Repeats 4–6 show the α-helical secondary structures that contain the matched glutamates. Repeats 7–9 show the full DHR protein backbone. Repeats 10–12 show the full backbone and all amino acid side chains. Repeats 13–15 show all atoms as spheres. Repeats 16–18 show the external surface of the protein. Reproduced with permission from Supplementary Ref. 1 (copyright Springer Nature, 2019).

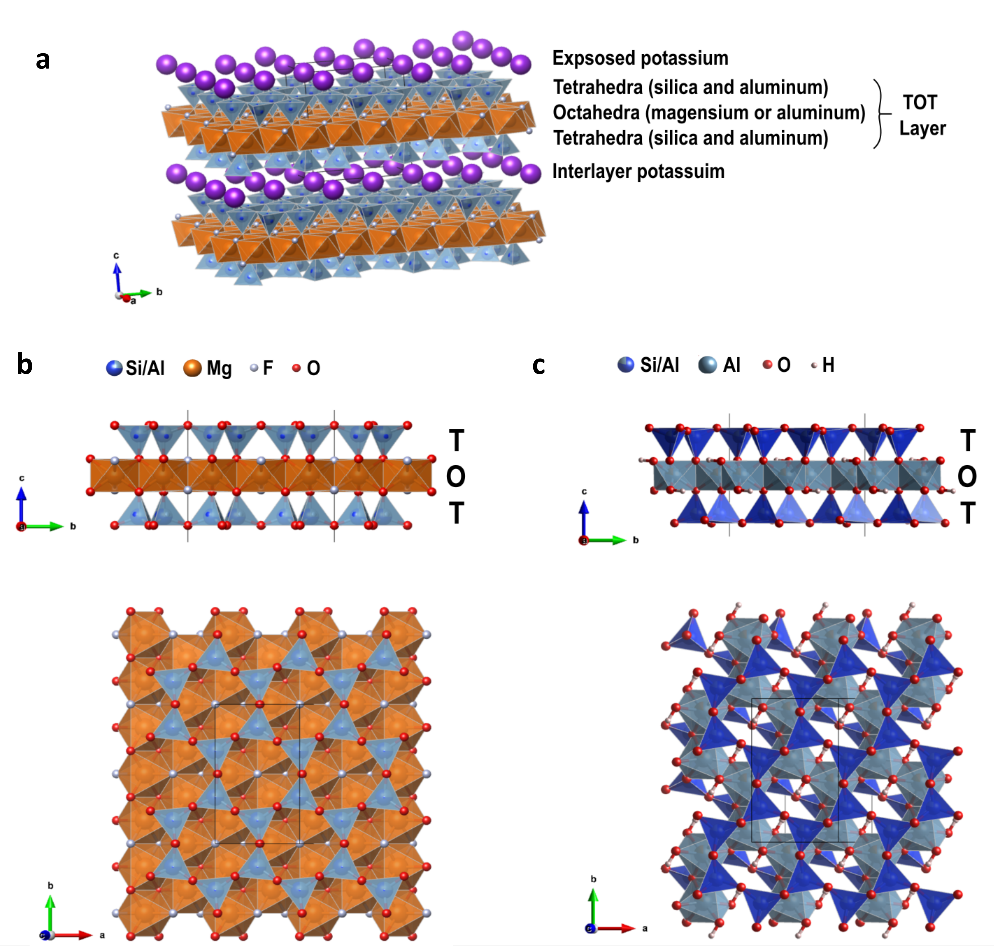

Supplementary Figure 2: (a) Micas are typically comprised of tetrahedral-octahedral-tetrahedral (TOT) sheets, bound together by a potassium interlayer. When cleaved, micas split at the interlayer, to expose the tetrahedral surface of the TOT sheets, and the free potassium ions that adsorb onto cavities of that surface. **(b)** Fluorphlogopite (i.e. f-mica) TOT sheets are shown here in cross-section and top view. The tetrahedral layer (blue) is comprised of silica tetrahedra with ¼ aluminum substitution. The octahedral layer is comprised of Mg octahedra (orange). All octahedral sites are filled, making this a trioctahedral mica. The tetrahedra form cavities on the surface from a nominally hexagonal lattice, but careful inspection shows that the TOT sheet of f-mica has trigonal symmetry, rather than true hexagonal symmetry. **(c)** Muscovite (i.e. m-mica) TOT sheets are shown here in cross-section and top view. The tetrahedral layers are still comprised of silica and alumina tetrahedral (dark blue), but the octahedral layer is comprised of Al octahedra (light blue). Only 2/3 of the octahedral sites are filled, making this a 'dioctahedral' mica. Although the cavities still form a nominally hexagonal lattice, the missing octahedral sites in m-mica cause

strong symmetry breaking of the TOT sheet. Couplings between the oxygens of the tetrahedral layer, and the hydroxyls attached to the octahedral layer cause distortions of the tetrahedral network. The direction along the hydroxyl (running from lower left to upper right, in the lower image) is unique from all other directions, and the sheet has no rotational symmetry.

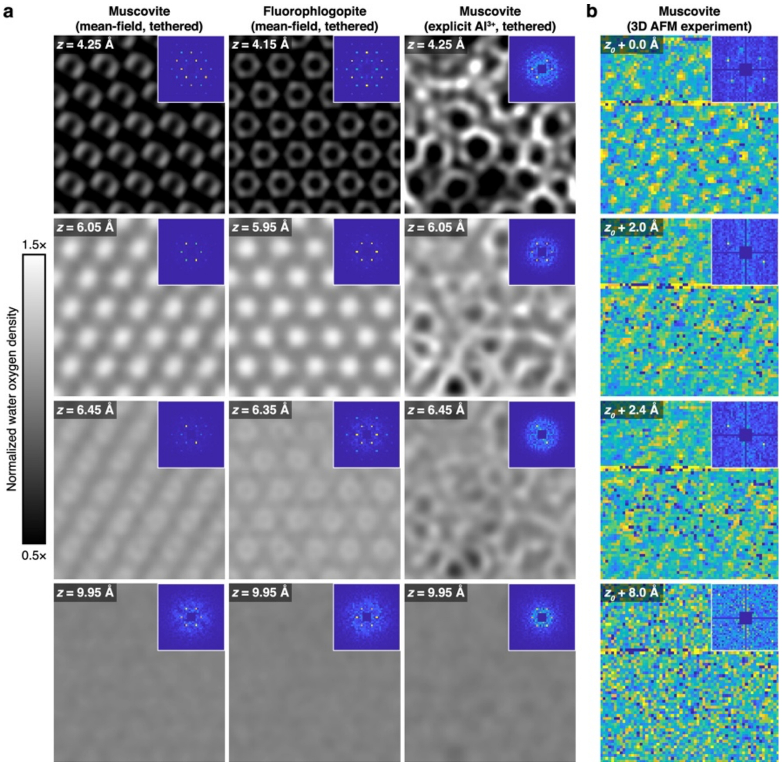

Supplementary Figure 3: Distinct solvent structure induced by the underlying mineral surface **(a)** Solution structure predicted by simulation. **(b)** Solution structure above muscovite mica corroborated experimentally by 3D Fast Force Mapping (3D FFM) (image reproduced with permission from Alberstein et al *J. Phys. Chem. Lett.* 2023, 14, 1, 80–87, copyright ACS, 2023).

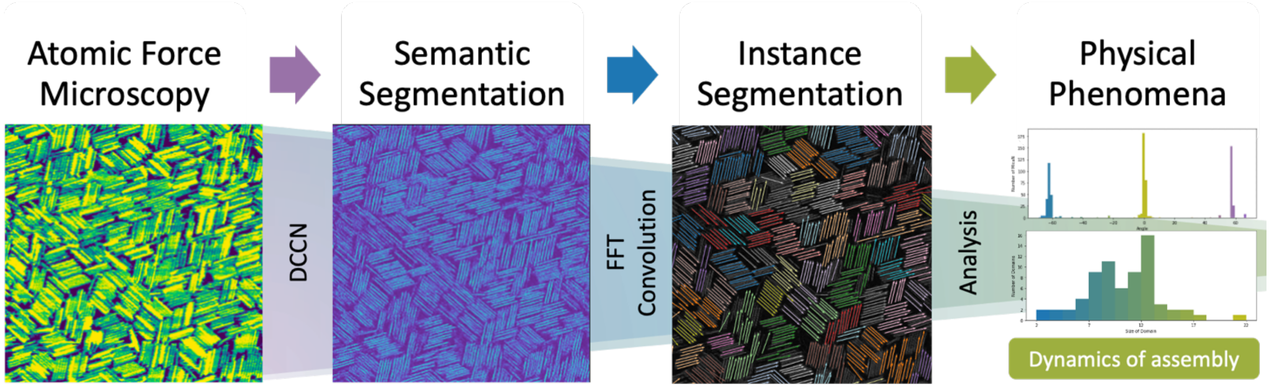

Supplementary Figure 4: Schematic describing the workflow of experimental analysis where the raw data is denoised using a deep convolutional neural network (DCCN). Individual rods are then recognized using FFT convolution which provides the position and angle of each rod for each frame in the experimental movies.

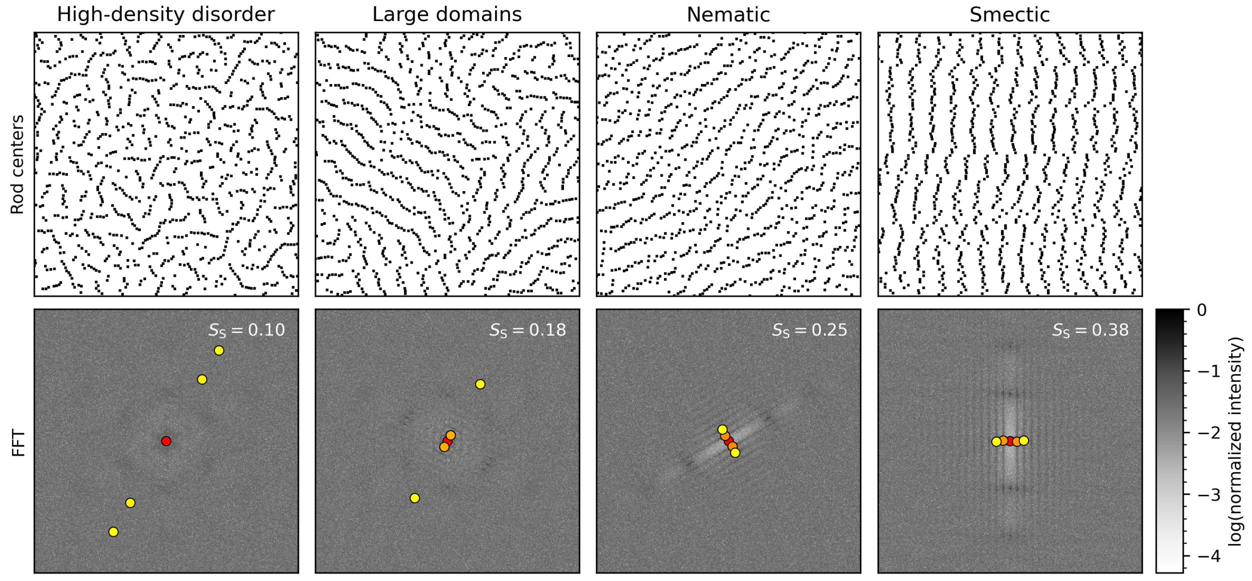

Supplementary Figure 5: The smectic order parameter for a given rod configuration is calculated by taking the Fourier transform (FFT) of the rod centers; the FFT intensities are normalized so that the maximum intensity is equal to one, and the second highest FFT intensity found exclusively within thin “strips” of pixels passing through the zero-frequency component and having rotation angles of 0°, 60°, and 120° then serves as the smectic order parameter. To illustrate this process, the top row of the figure shows images of the rod centers for four distinct rod configurations. The bottom row shows the FFT of these rod-center images with circles indicating the five locations in the relevant FFT strip with the highest intensities; the circles are colored according to their intensity (yellow lowest, red highest). The (un-normalized) value of the smectic order parameter is provided at the top right of each FFT image. (The smectic order parameters in the main text are normalized so that the highest value obtained across the simulations or experiments, separately, is equal to one.) Source data are provided as a Source Data file.

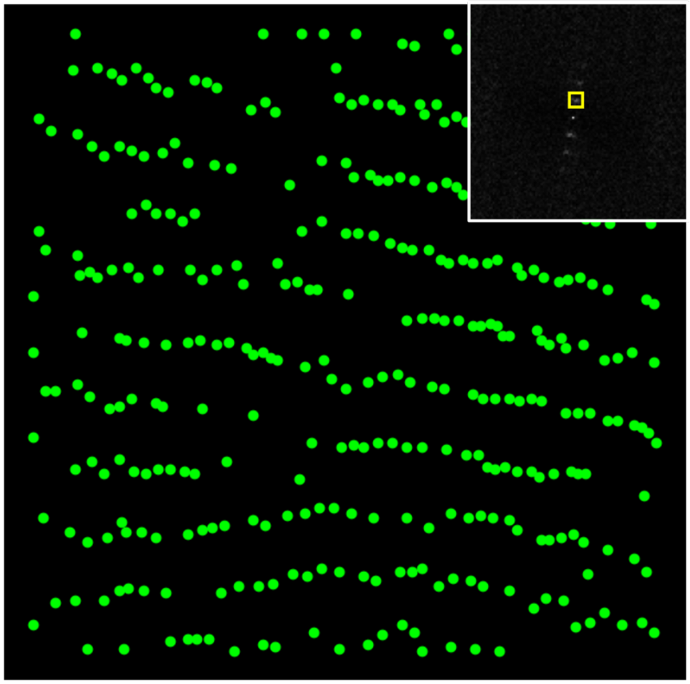

Supplementary Figure 6: Example of the process to obtain the smectic order parameter from experimental data showing a plot of the rod centers from the 650th frame of the experimental video obtained on m-mica (left) and the FFT of the rod centers with the window containing the detected peak which corresponds to the distance between rows of rods marked in yellow (inset).

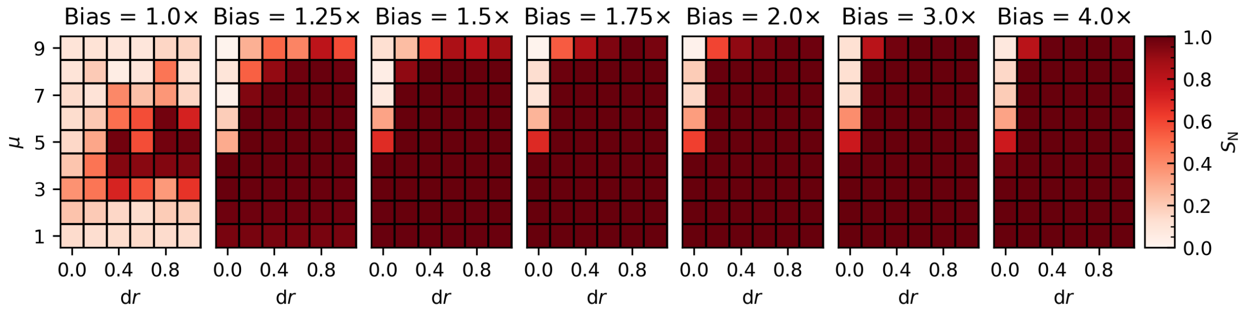

Supplementary Figure 7: Average nematic order parameter, S, for an ensemble of Monte Carlo simulations with chemical potentials, μ, ranging from 1.0 to 9.0; rod mobilities, dr, ranging from 0.0 to 1.0; and for which the horizontally oriented rods have been biased to be up to four times as energetically favorable as rods in the other two orientations. The baseline rod energy is set at -2 k_BT, and all simulations use a rod aspect ratio $\ell = 7$. Each box represents a single Monte Carlo simulation. The nematic order parameter for a given snapshot is calculated according to Eq. (1) of the main text; plotted values correspond to the order parameter averaged over the last fifth of the Monte Carlo simulation. A snapshot of the final rod configuration for each of the simulations in the ensemble is given in Supplementary Figs. 14-18, 21, 25. Source data are provided as a Source Data file.

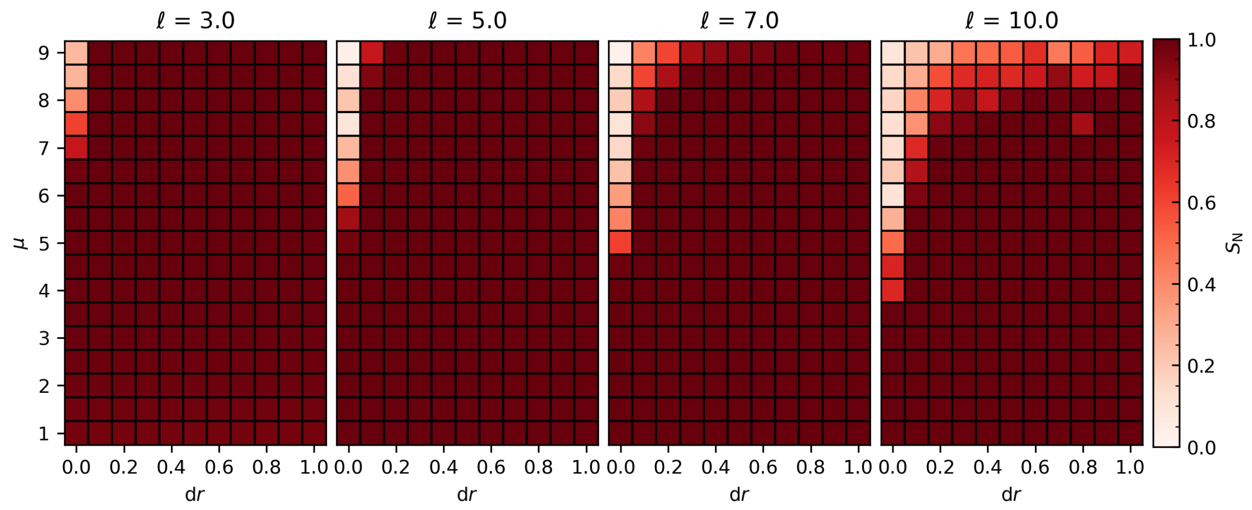

Supplementary Figure 8: Average nematic order parameter for an ensemble of Monte Carlo simulations with chemical potentials, μ, ranging from 1.0 to 9.0; rod mobilities, dr, ranging from 0.0 to 1.0; rod aspect ratios ℓ of 3, 5, 7, and 10; and for which the horizontally oriented rods have been biased to be twice as energetically favorable as rods in the other two orientations. The baseline rod energy is set at -2 k_BT. Each box represents a single Monte Carlo simulation. The nematic order parameter for a given snapshot is calculated according to Eq. (1) of the main text; plotted values correspond to the order parameter averaged over the last fifth of the Monte Carlo simulation. A snapshot of the final rod configuration for each of the simulations in the ensemble is given in Supplementary Figs. 23-26. Soure data are provided as a Source Data file.

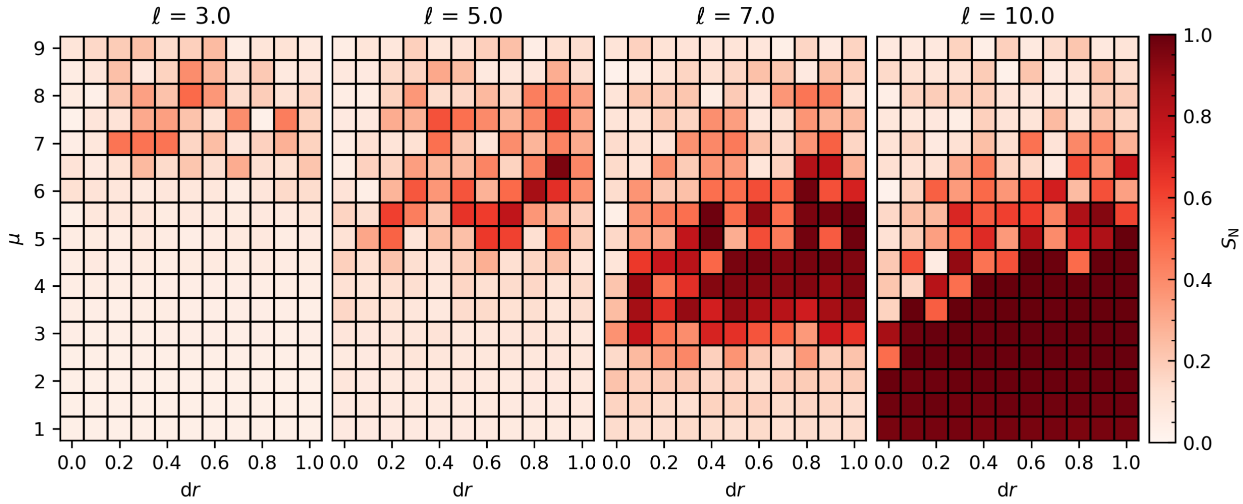

Supplementary Figure 9: Average nematic order parameter for an ensemble of Monte Carlo simulations with chemical potentials, μ, ranging from 1.0 to 9.0; rod mobilities, dr, ranging from 0.0 to 1.0; rod aspect ratios ℓ of 3, 5, 7, and 10; and for which all three rod orientations are equally favorable. Each box represents a single Monte Carlo simulation. The nematic order parameter for a given snapshot is calculated according to Eq. (1) of the main text; plotted values correspond to the order parameter averaged over the last fifth of the Monte Carlo simulation. A snapshot of the final rod configuration for each of the simulations in the ensemble is given in Supplementary Figs. 19-22. Source data are provided as a Source Data file.

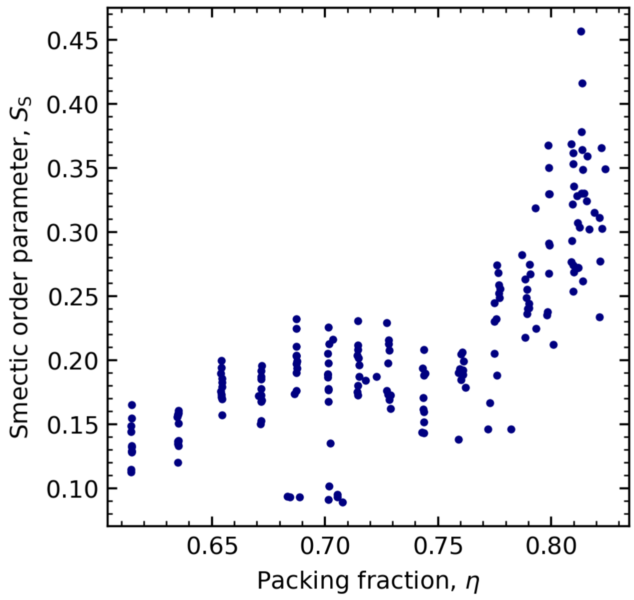

Supplementary Figure 10: Relationship between smectic order parameter, S_S, and packing fraction, η, for the ensemble of Monte Carlo simulations with rod aspect ratio ℓ = 7 and with the horizontal rod orientation twice as energetically favorable as the other two orientations (the baseline rod energy is -2 k_BT). Smectic order achieves a local maximum at a packing fraction of approximately 0.70, momentarily decreases, and then rapidly increases with packing fractions above approximately 0.76. Source data are provided as a Source Data file.

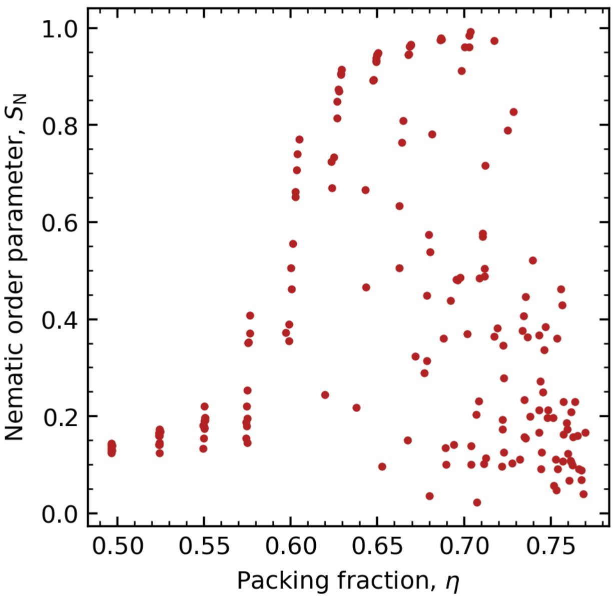

Supplementary Figure 11: Relationship between nematic order parameter, S_N, and packing fraction, η, for the ensemble of Monte Carlo simulations with rod aspect ratio $\ell = 7$ and with all rod orientations equally favorable. Nematic order peaks at a packing fraction of approximately 0.70. Source data are provided as a Source Data file.

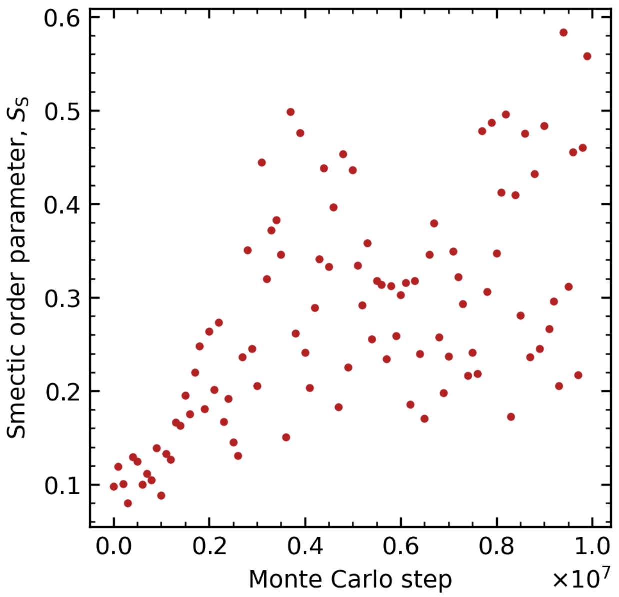

Supplementary Figure 12: Evolution of the smectic order parameter, S_S, over the course of a Monte Carlo Simulation with $\mu = 8.0$, dr = 0.6, $\ell = 7$, and where one rod orientation is twice as energetically favorable as the other two. Source data are provided as a Source Data file.

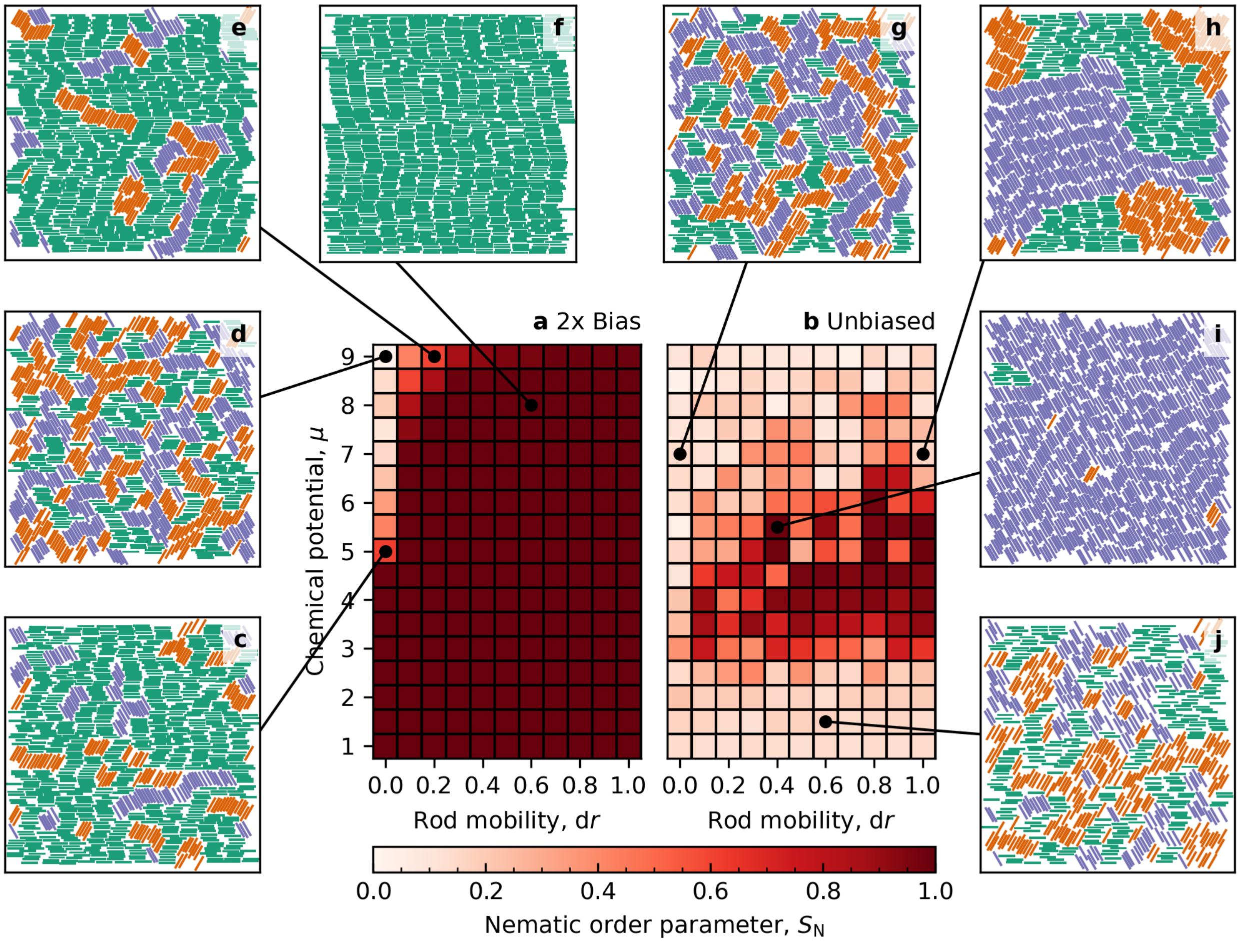

Supplementary Figure 13: The nematic order parameters for a collection of Monte Carlo simulations of hard rods with aspect ratio $\ell = 7$ are presented in grids, where each box in the grid represents a single simulation defined its chemical potential and rod mobility; the coloring indicates the value of the nematic order parameter. The nematic order parameter for a given snapshot is calculated according to Eq. (1) of the main text; plotted values correspond to the order parameter averaged over the last fifth of the Monte Carlo simulation. Separate grids are plotted for simulation collections where all three rod orientations are equally favorable (**b**, "Unbiased"), and where the horizontal rods are twice as energetically favorable as the other orientations **(a,** "2x Bias"). Several select simulations are annotated with a snapshot of the final rod configuration (**c-j**); in the provided snapshots, rods of the same orientation share the same color. Source data are provided as a Source Data file.

Additional Monte Carlo Simulations Figures (A high-resolution version of each figure is provided as Supplementary Data)

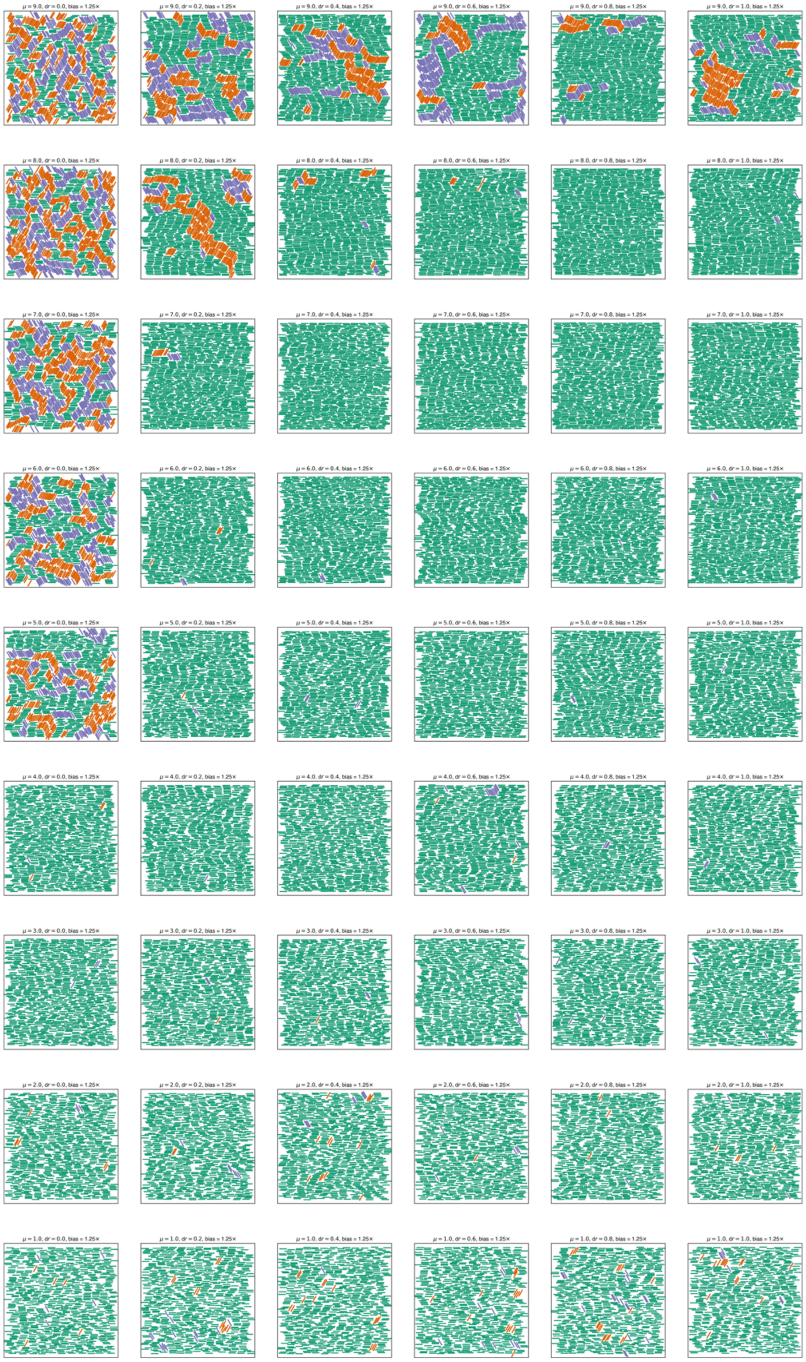

Supplementary Figure 14: Snapshots of the final rod configurations from Monte Carlo simulations with different chemical potentials and rod mobilities where the horizontal rods are 1.25x as energetically favorable as rods in other orientations: the baseline rod energy is -2 $k_B T$, and the horizontal rods have energy -2.5 $k_B T$. All rods have aspect ratio $\ell = 7$. Rods of the same orientation share the same color.

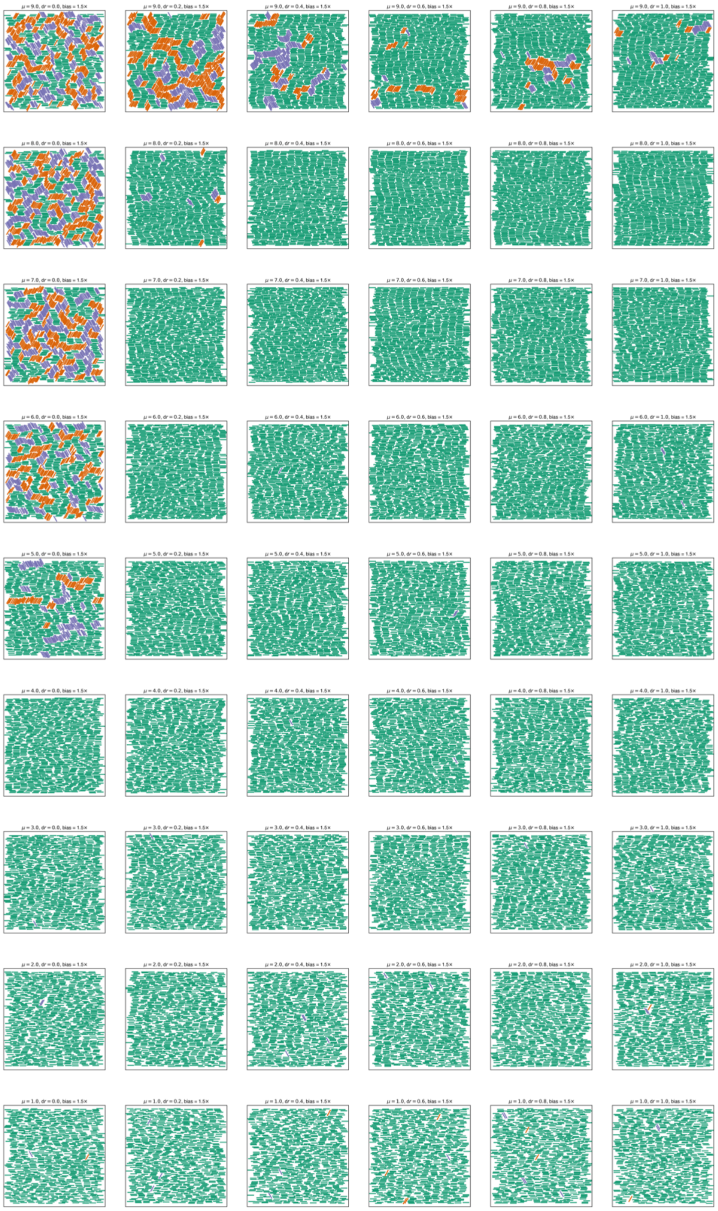

Supplementary Figure 15: Snapshots of the final rod configurations from Monte Carlo simulations with different chemical potentials and rod mobilities where the horizontal rods are 1.5x as energetically favorable as rods in other orientations: the baseline rod energy is -2 k_BT, and the horizontal rods have energy -3 k_BT. All rods have aspect ratio ℓ = 7. Rods of the same orientation share the same color.

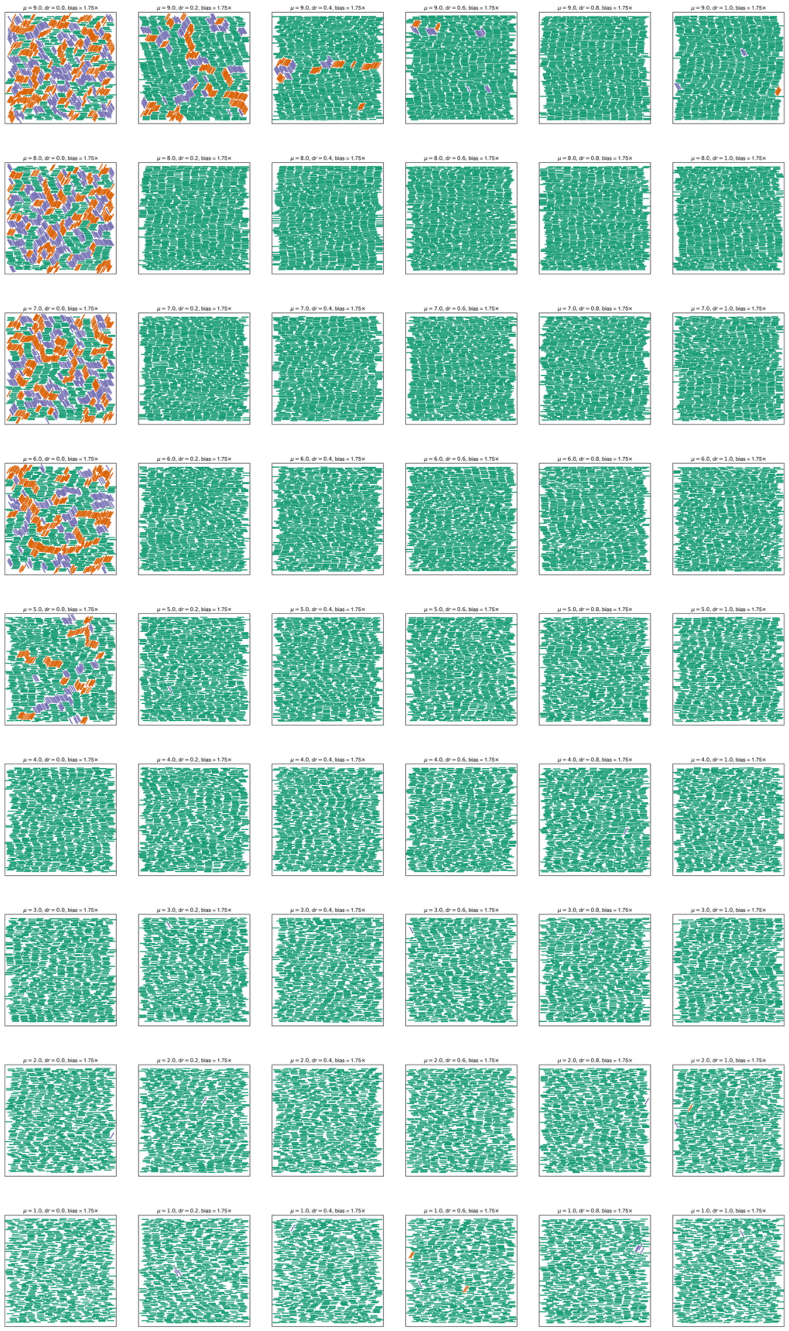

Supplementary Figure 16: Snapshots of the final rod configurations from Monte Carlo simulations with different chemical potentials and rod mobilities where the horizontal rods are 1.75x as energetically favorable as rods in other orientations: the baseline rod energy is -2 k_BT, and the horizontal rods have energy -3.5 k_BT. All rods have aspect ratio $\ell = 7$. Rods of the same orientation share the same color.

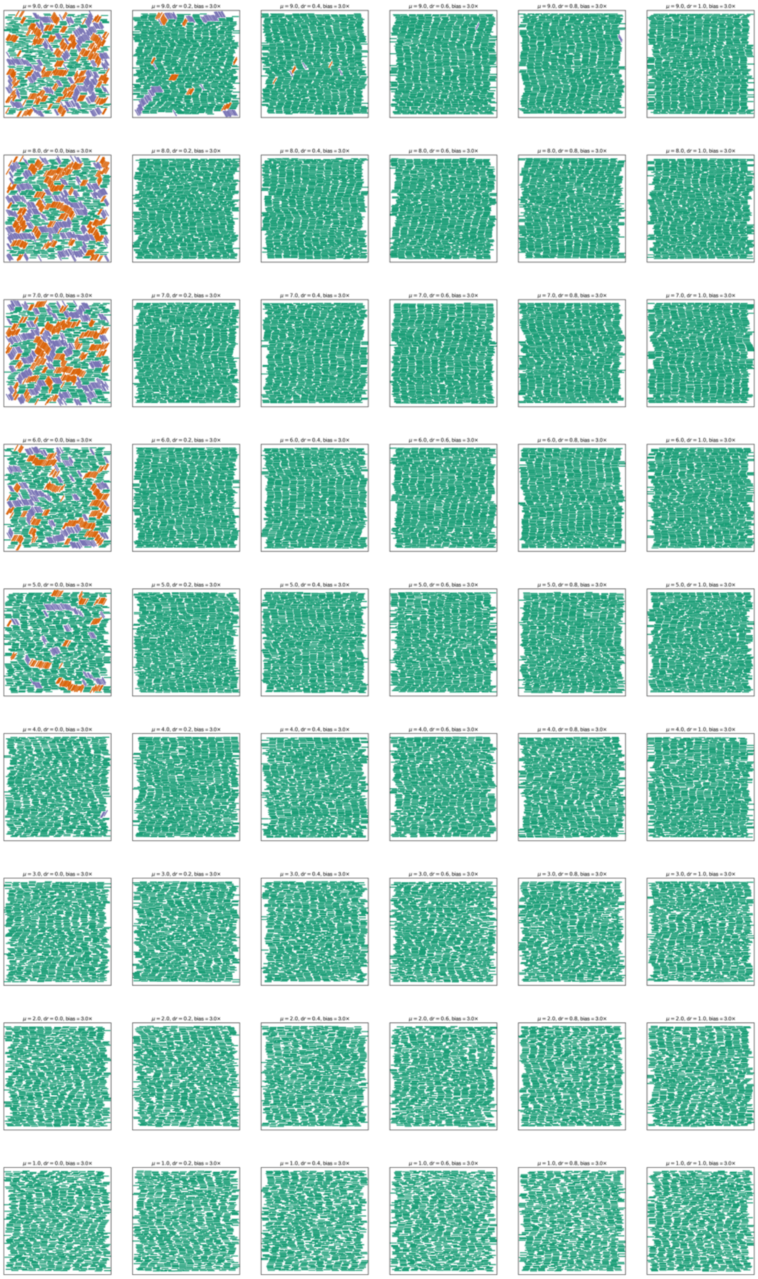

Supplementary Figure 17: Snapshots of the final rod configurations from Monte Carlo simulations with different chemical potentials and rod mobilities where the horizontal rods are 3x as energetically favorable as rods in other orientations: the baseline rod energy is -2 k_BT, and the horizontal rods have energy -6 k_BT. All rods have aspect ratio $\ell = 7$. Rods of the same orientation share the same color.

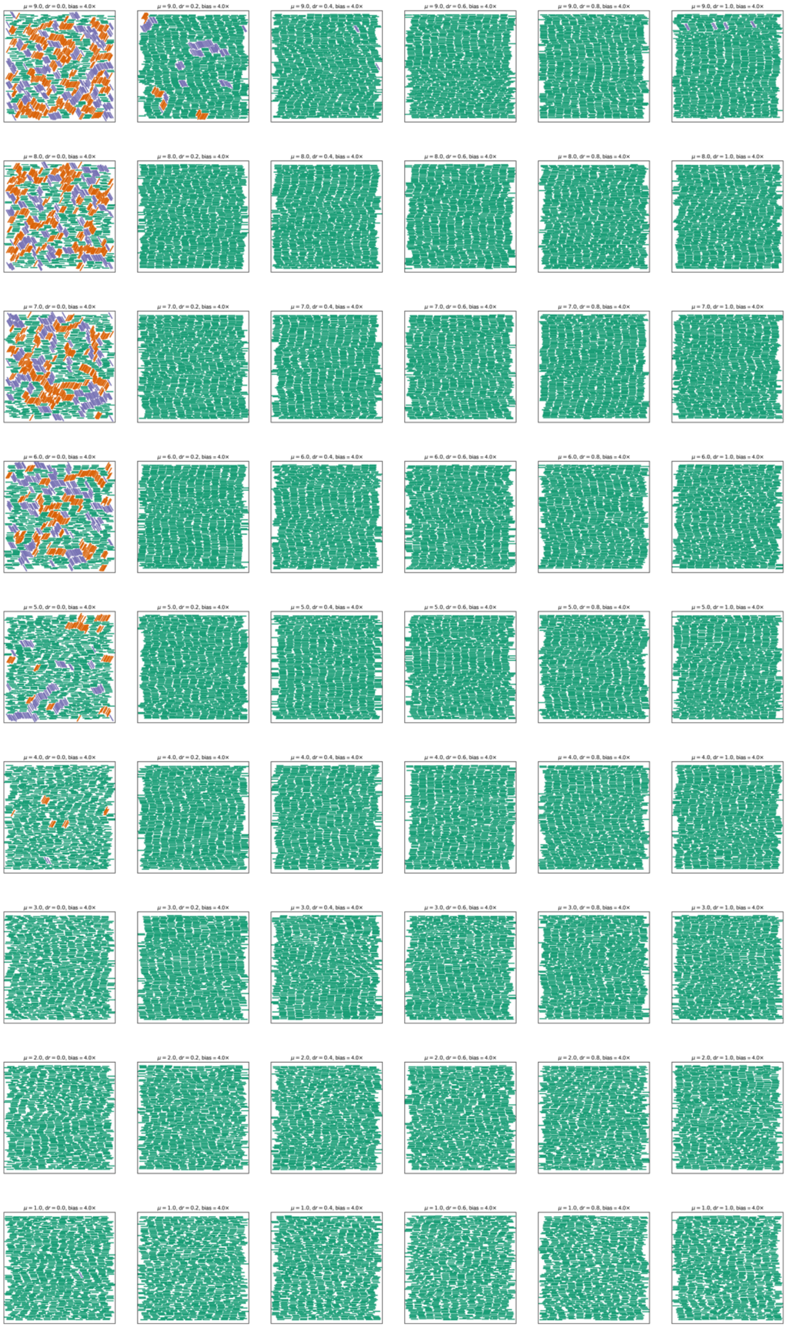

Supplementary Figure 18: Snapshots of the final rod configurations from Monte Carlo simulations with different chemical potentials and rod mobilities where the horizontal rods are 4x as energetically favorable as rods in other orientations: the baseline rod energy is -2 k_BT, and the horizontal rods have energy -8 k_BT. All rods have aspect ratio $\ell = 7$. Rods of the same orientation share the same color.

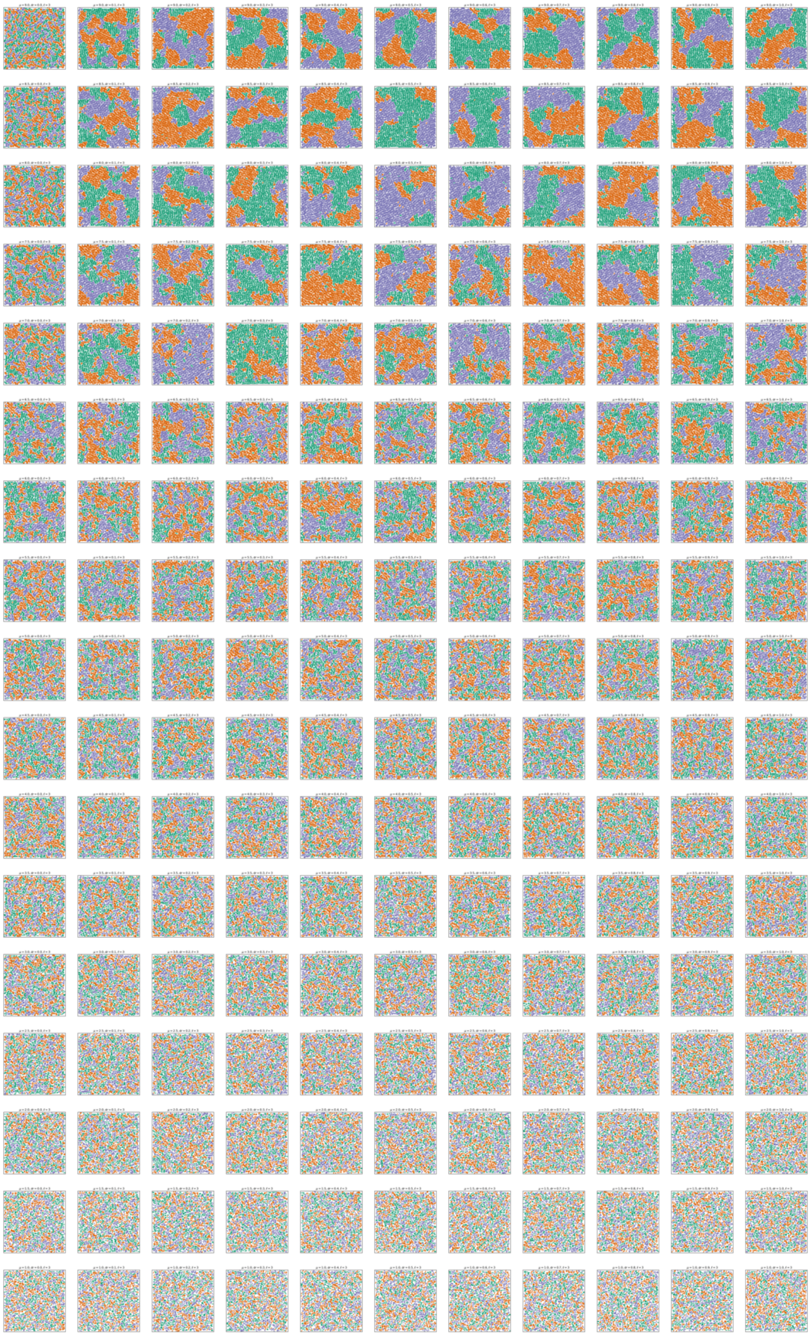

Supplementary Figure 19: Snapshots of the final rod configurations from Monte Carlo simulations with different chemical potentials and rod mobilities where all three rod orientations are equally favorable, and all rods have aspect ratio $\ell = 3$. Rods of the same orientation share the same color.

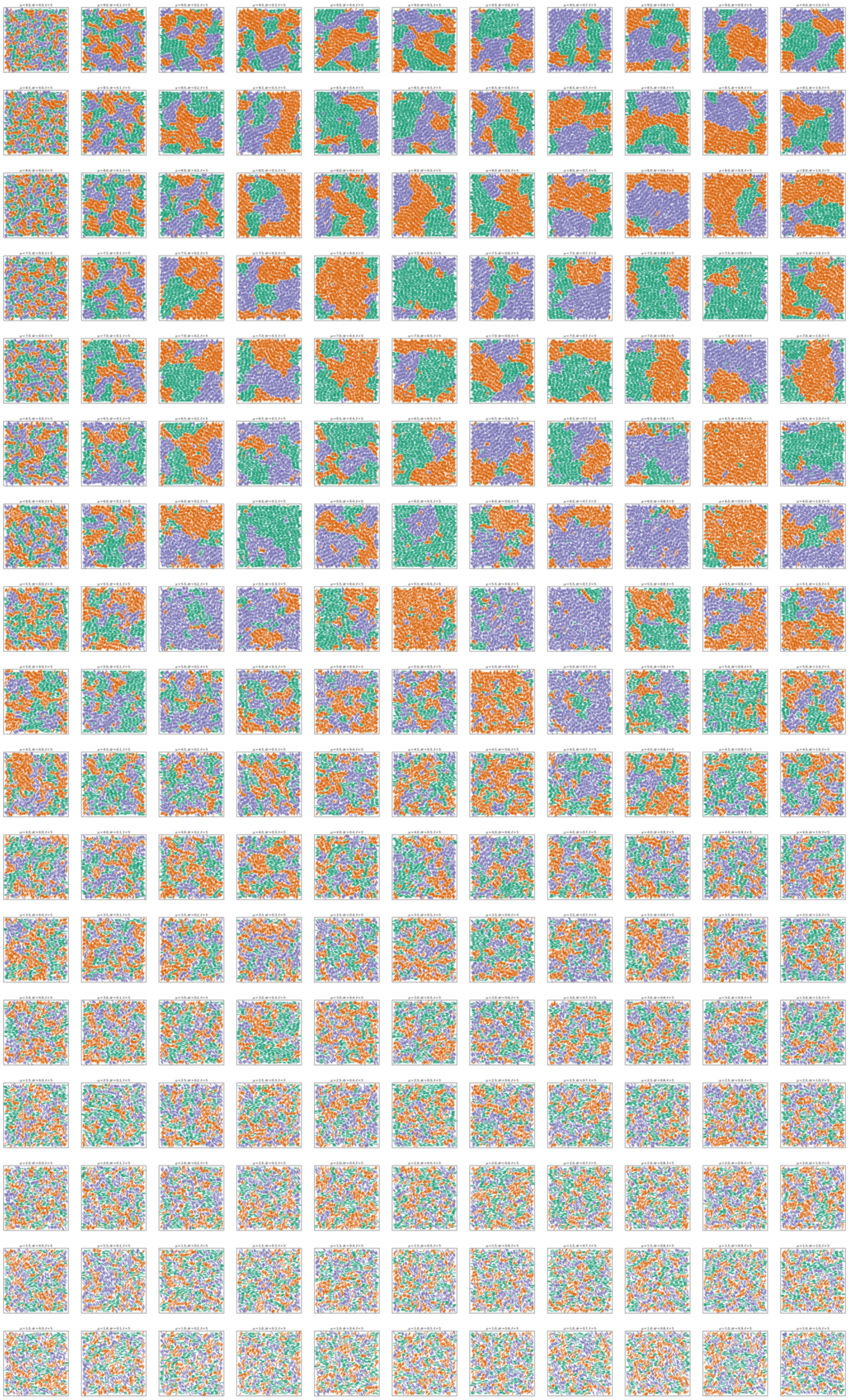

Supplementary Figure 20: Snapshots of the final rod configurations from Monte Carlo simulations with different chemical potentials and rod mobilities where all three rod orientations are equally favorable, and all rods have aspect ratio $\ell = 5$. Rods of the same orientation share the same color.

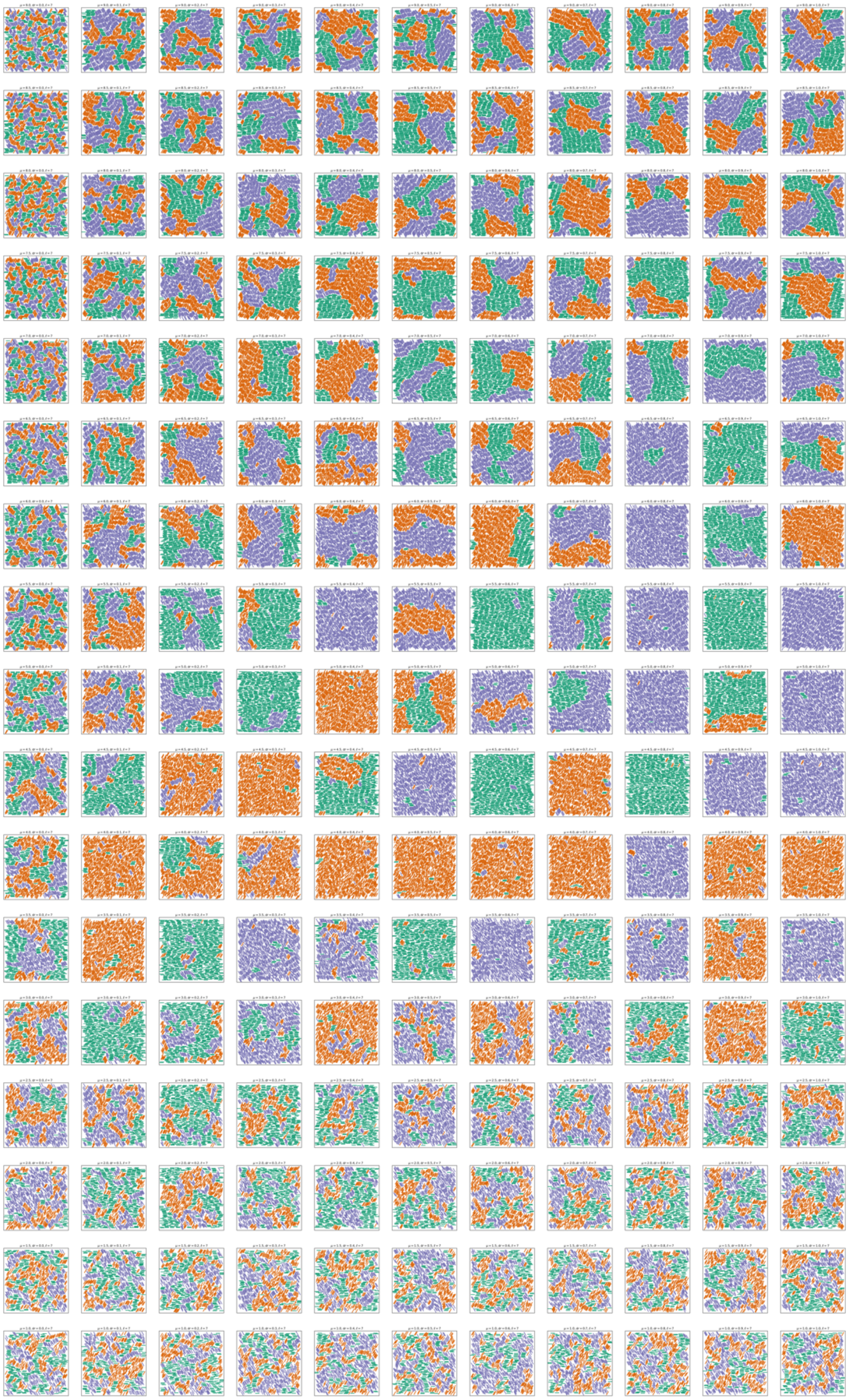

Supplementary Figure 21: Snapshots of the final rod configurations from Monte Carlo simulations with different chemical potentials and rod mobilities where all three rod orientations are equally favorable, and all rods have aspect ratio $\ell = 7$. Rods of the same orientation share the same color.

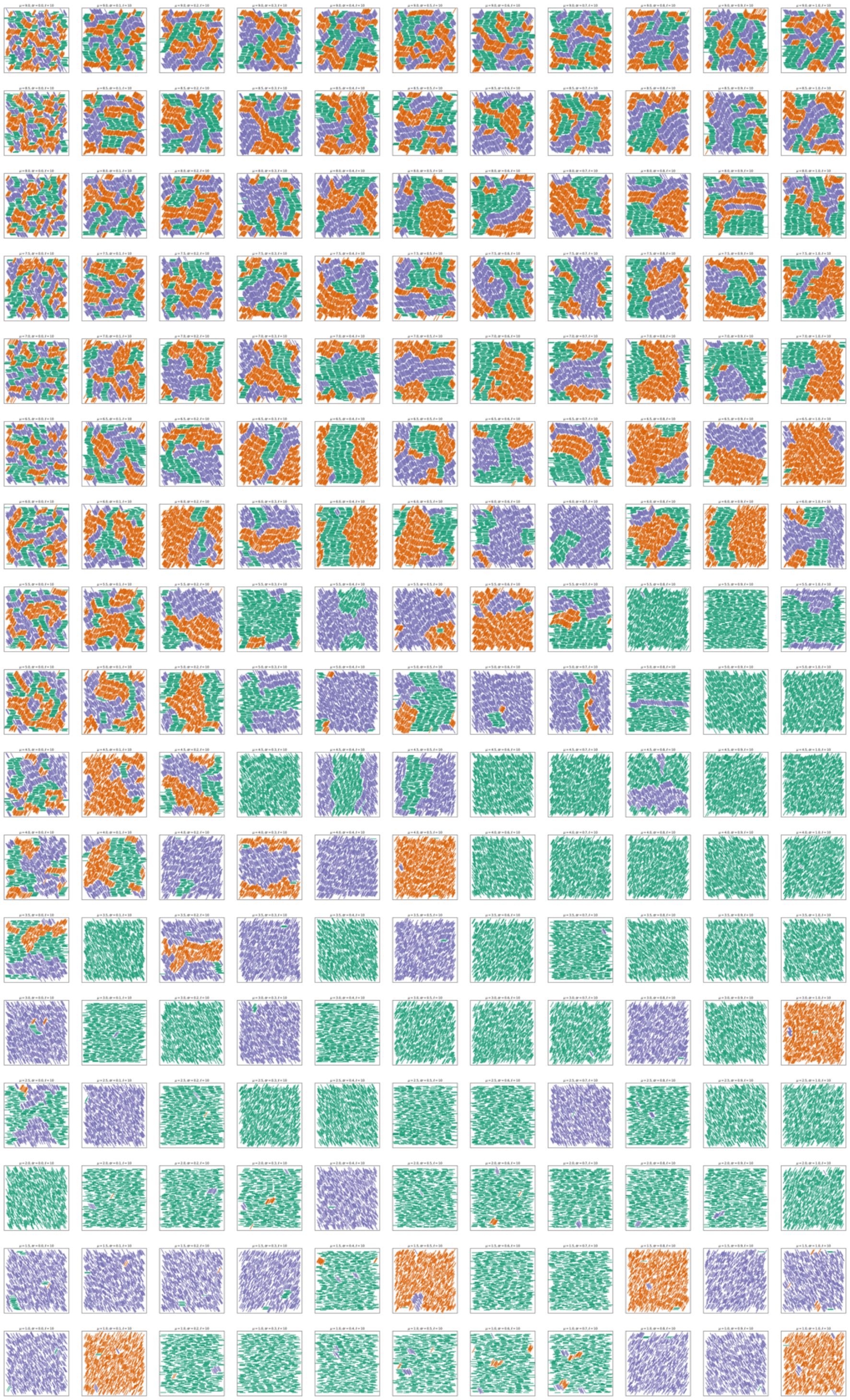

Supplementary Figure 22: Snapshots of the final rod configurations from Monte Carlo simulations with different chemical potentials and rod mobilities where all three rod orientations are equally favorable, and all rods have aspect ratio $\ell = 10$. Rods of the same orientation share the same color.

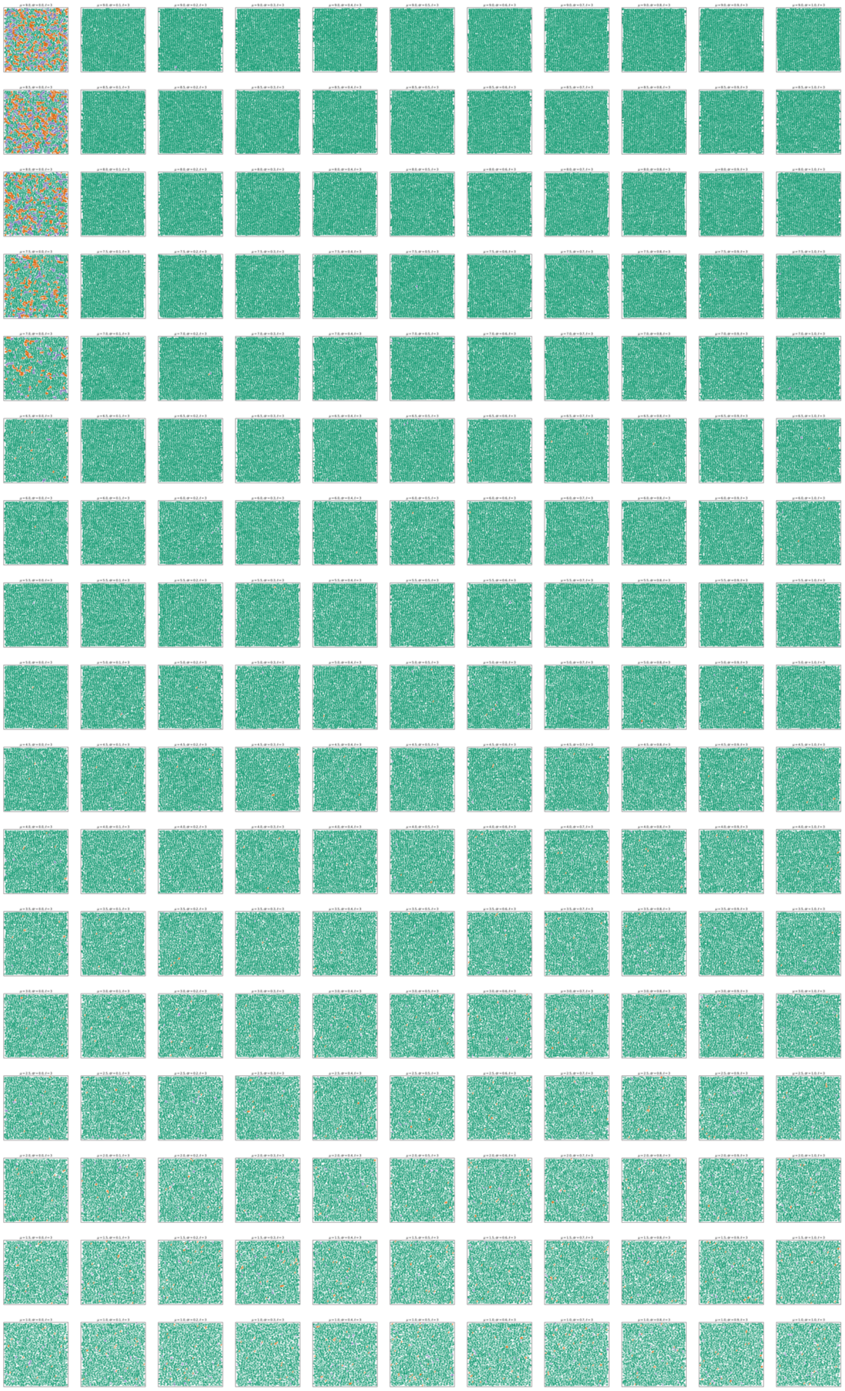

Supplementary Figure 23: Snapshots of the final rod configurations from Monte Carlo simulations with different chemical potentials and rod mobilities where the horizontal rods are twice as energetically favorable as the other orientations and all rods have aspect ratio $\ell = 3$. Rods of the same orientation share the same color.

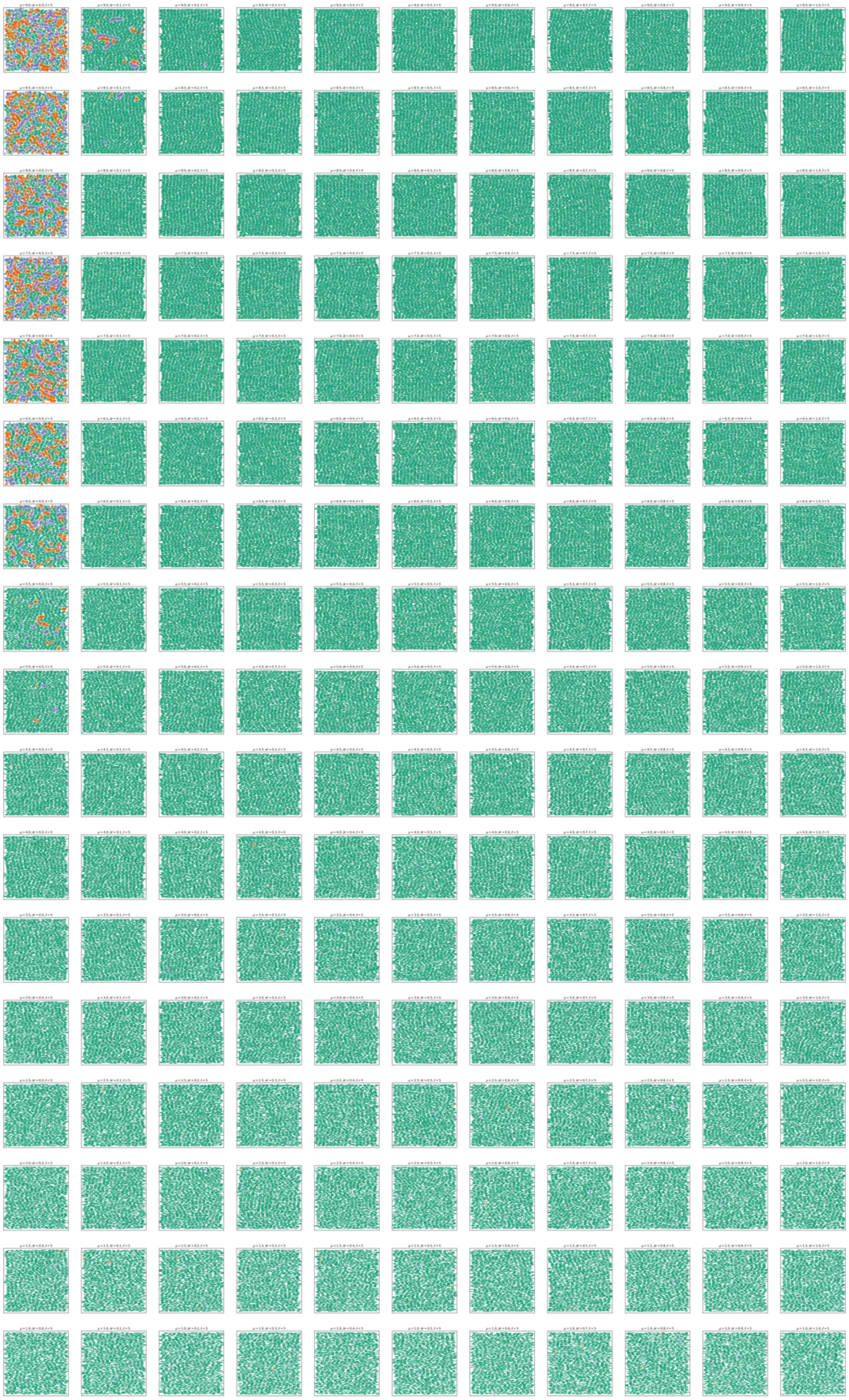

Supplementary Figure 24: Snapshots of the final rod configurations from Monte Carlo simulations with different chemical potentials and rod mobilities where the horizontal rods are twice as energetically favorable as the other orientations and all rods have aspect ratio $\ell = 5$. Rods of the same orientation share the same color.

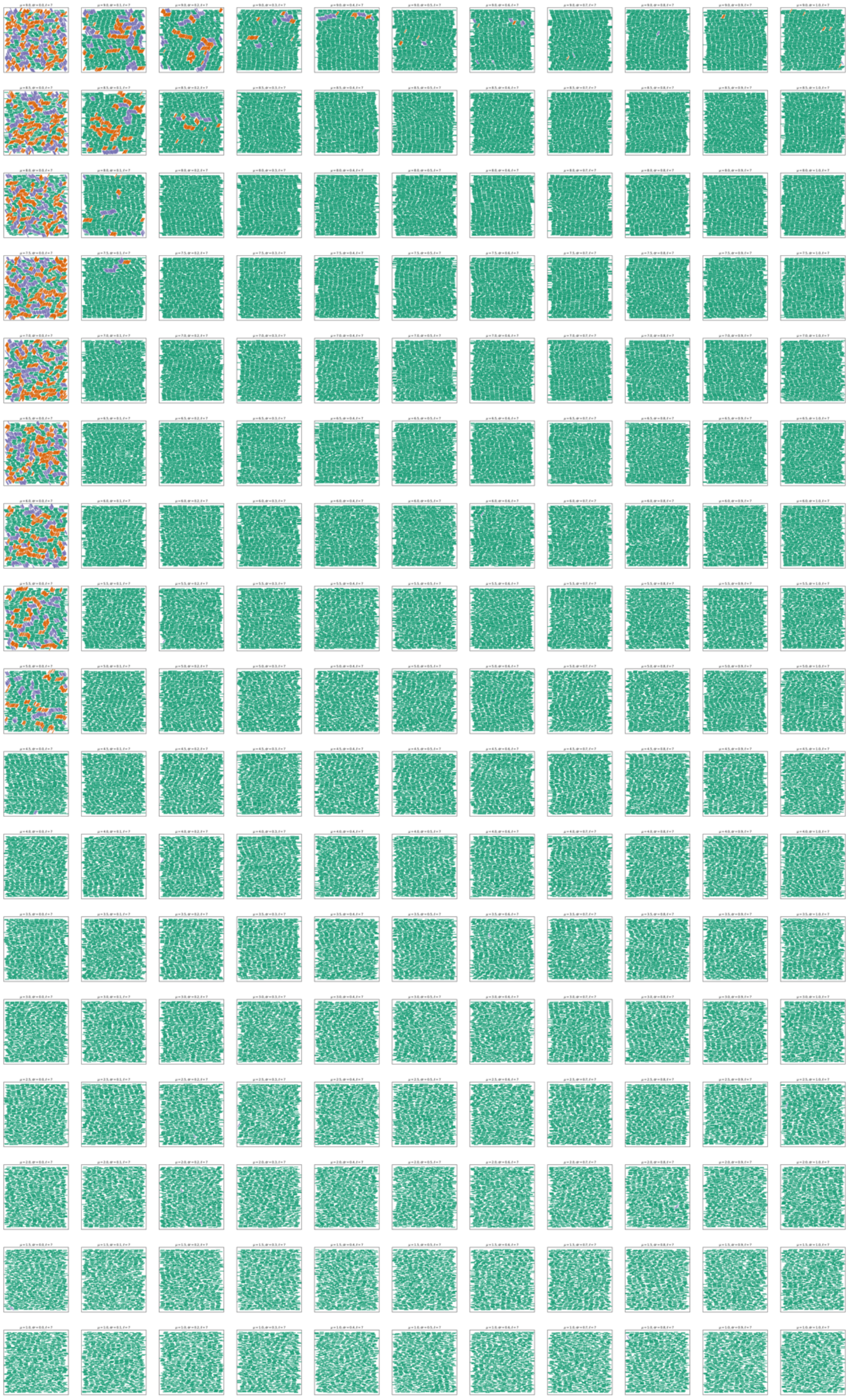

Supplementary Figure 25: Snapshots of the final rod configurations from Monte Carlo simulations with different chemical potentials and rod mobilities where the horizontal rods are twice as energetically favorable as the other orientations and all rods have aspect ratio $\ell = 7$. Rods of the same orientation share the same color.

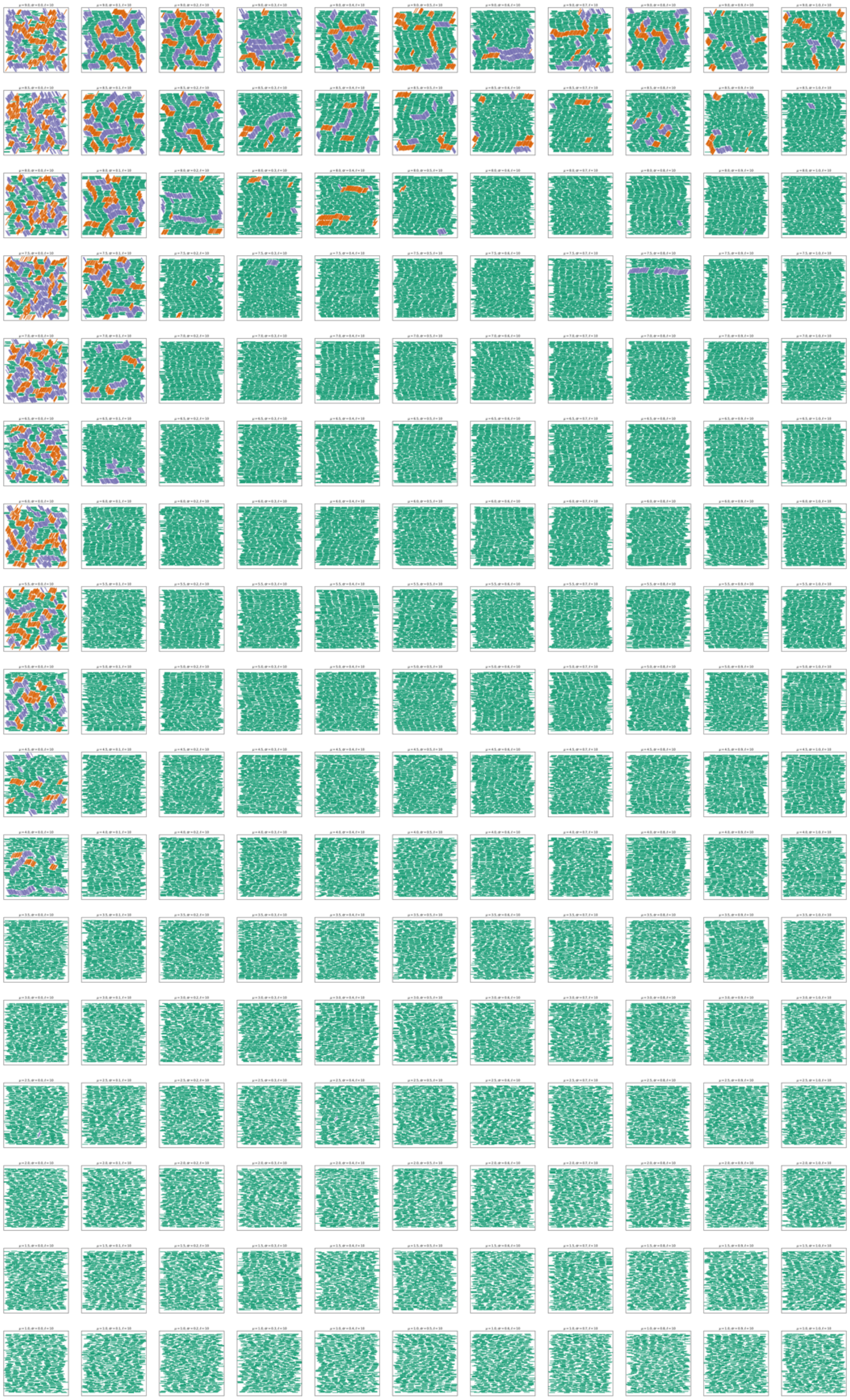

Supplementary Figure 26: Snapshots of the final rod configurations from Monte Carlo simulations with different chemical potentials and rod mobilities where the horizontal rods are twice as energetically favorable as the other orientations and all rods have aspect ratio $\ell = 10$. Rods of the same orientation share the same color.

Supplementary references

1. Pyles, H., Zhang, S., De Yoreo, J. J. & Baker, D. Controlling protein assembly on inorganic crystals through designed protein interfaces. *Nature* **571**, 251–256 (2019).

2. Kalinin, S. V. *et al.* Disentangling Rotational Dynamics and Ordering Transitions in a System of Self-Organizing Protein Nanorods via Rotationally Invariant Latent Representations. *ACS Nano* **15**, 6471–6480 (2021).

3. *Machine Learning and Artificial Intelligence in Chemical and Biological Sensing*. (Elsevier, 2024). doi:10.1016/C2023-0-00229-1.

4. Ziatdinov, M., Ghosh, A., Wong, C. Y. & Kalinin, S. V. AtomAI framework for deep learning analysis of image and spectroscopy data in electron and scanning probe microscopy. *Nat Mach Intell* **4**, 1101–1112 (2022).
