# Supplementary material for: Impact of solvent forces and broken symmetry on the assembly of designed proteins at a liquid-solid interface
Source: Nat Commun. 2026 Mar 13;17:2446. doi: 10.1038/s41467-026-69170-0 (PMC12987978; doi:10.1038/s41467-026-69170-0)
Supplement: Supplementary file 2 — Description of Additional Supplementary Files [file 41467_2026_69170_MOESM2_ESM.pdf]

File Name: Supplementary Movie 1
Description: (A) HS-AFM video showing the translational motion of protein rods and their assembly on muscovite mica in 3 M KCl. Scan size: 200 nm. (B) Machine learning semantic segmentation. (C) Machine learning -recognized centers of rods. (D) FFT image.

File Name: Supplementary Movie 2
Description: (A) HS-AFM video showing the translational motion of protein rods and their assembly on fluorophlogopite mica in 3 M KCl. Scan size: 200 nm. (B) Machine learning semantic segmentation. (C) Machine learning-recognized centers of rods. (D) FFT image.

File Name: Supplementary Data 1
Description: High-resolution versions of Supplemental Figs. 14 to 26.

File Name: Supplementary Data 2
Description: Machine learning code for the HS-AFM video analysis.

File Name: Source Data
Description: Source Data
